# Deep Venous Thrombosis in Patients with Erythema Nodosum Leprosum in the Use of Thalidomide and Systemic Corticosteroid in Reference Service in Belo Horizonte, Minas Gerais

**DOI:** 10.1155/2019/8181507

**Published:** 2019-06-19

**Authors:** Luiz Alberto Bomjardim Pôrto, Maria Aparecida de Faria Grossi, Edilamar Silva de Alecrim, Marcus Henrique de Souza Brito Xavier, Frederico Paiva e Silva, Amalia Sathler Pires, Fabianny Sanglard da Silva, Sandra Lyon

**Affiliations:** ^1^Dermatology Service Eduardo de Menezes Hospital, FHEMIG, Belo Horizonte, Minas Gerais, Brazil; ^2^Secretary of State for Health, Belo Horizonte, Minas Gerais, Brazil; ^3^Dermatology Service Alberto Cavalcante Hospital, Belo Horizonte, Minas Gerais, Brazil

## Abstract

**Introduction:**

Erythema nodosum leprosum (ENL) is a type of lepra reaction treated with corticosteroids and thalidomide, but this association increases the risk of deep venous thrombosis (DVT).

**Objective:**

To report cases of ENL with DVT in the use of thalidomide/corticosteroid associated.

**Methodology:**

The study was conducted between December 2015 and December 2016 at the Eduardo de Menezes Hospital (HEM-FHEMIG).

**Results:**

A clinical case series of 16 patients, eight from HEM-FHEMIG and eight from the literature. DVT occurred on 4 continents, mainly in adults and men. All patients were multibacillary; four people had pulmonary embolism (PE); there were 11 unilateral and five bilateral DVT cases; 12 cases were proximal, two distal, and two unspecified. Pharmacological thromboprophylaxis was used on two individuals. Outcome after DVT, 14 patients improved, one had sequelae, and one died.

**Discussion:**

DVT increased in association with thalidomide/corticosteroid in multiple myeloma, but this complication is poorly described in ENL. In proximal DVT, there was a greater risk of PE and sequelae venous insufficiency. After DVT, start anticoagulation. ASA 100mg/day as prophylaxis for DVT in case of this drug association in ENL is recommended.

**Conclusion:**

The article illustrates the incidence increase of DVT because of the thalidomide/corticosteroid combination in ENL. When this association is necessary, use ASA 100mg/day as prophylaxis.

## 1. Introduction

According to the World Health Organization (WHO), there were 210671 new cases of leprosy, in 2017 which represents a detection rate of 2.77 cases per 100 thousand inhabitants. Brazil has high burden for an illness, being the second with the highest number of new cases registered in the world [1].

Leprosy is a chronic infection caused by Mycobacterium leprae that primarily attacks the skin and peripheral nerves. The clinical presentation depends on the genetically determined cellular immunity of the patient. Accordingly, the disease is often categorized as multibacillary or paucibacillary (lepromatous or tuberculoid) which determines the type of therapeutic scheme [2].

Leprosy reactions are acute inflammatory episodes that may occur multiple times during the course of the disease or after treatment, manifesting as type 1 reaction or type 2 reaction known as erythema nodosum leprosum (ENL), whose treatment of choice is thalidomide [3–5]. The use of thalidomide associated with corticosteroids, cyclophosphamide, and doxorubicin may increase thromboembolic risk [6]. There are studies associating a 10% increase in the risk of deep venous thrombosis (DVT) with thalidomide in combination with systemic corticosteroids [5, 7].

Thrombosis is linked to an imbalance of the fibrinolytic system and has as risk factors previous diseases, use of medication and alteration, of pro- or anticoagulant factors [8].

In the literature review, eight patients with leprosy erythema nodosum treated with thalidomide associated with systemic corticosteroids presented thrombotic events [5–12].

In this series, we present eight cases of DVT in individuals with leprosy and type 2 reaction.

## 2. Objective

The present study aims to report clinical cases of patients with ENL with the outcome of DVT during the use of thalidomide and systemic corticosteroids.

## 3. Methodology

The study was conducted between December 2015 and December 2016, at the Hospital Eduardo de Menezes of the Hospital Foundation of the State of Minas Gerais (HEM-FHEMIG). HEM-FHEMIG is a Regional Reference Center for Health Dermatology and Infectious Diseases [13].

The preparation of this series of cases took into account anamnesis, clinical examination, and propaedeutic exams. A review of the literature on “deep vein thrombosis" related to the use of systemic corticosteroid therapy and thalidomide was performed. The research was done in publications made after 2004, in English and Portuguese, using the following health sciences descriptors: Pubmed, google academic, LILLACS, and Epistemonikos. There were few manuscripts on this subject, most of which were case reports.

Patient images were stored with appropriate descriptions in the DermatologiaWeb Software and later selected for the article. The DermatologiaWeb system is used for photographic documentation of patients in a digital virtual way. It includes photo storage functionality and the comparison function of the most recent images with the oldest images of the same lesion or of the same part of the body [14, 15].

## 4. Clinical Case Series

Table 1 presents a series of 16 clinical cases of DVT in ENL patients in the period of thalidomide and corticosteroid use, of which eight were followed in the HEM-FHEMIG and the other cases coming from reports of cases found in the English and Portuguese language literature. Thus, it is observed that such complication was described in in North America (1), South America (9), Europe (2), and Asia (4).

The patients' age range was 33 to 75 years (mean of 48.3 years). There was a predominance of males in the proportion of 12:4. All 16 patients were multibacillary in the lepromatous leprosy (9), borderline (3), and borderline lepromatous (3) forms and were not subclassified (1). Three patients had HIV-AIDS, one had cutaneous leishmaniasis, one had hypertension and dyslipidemia, one had diabetes, and one had diabetes, asthma, alcoholism, and a mood disorder.

Table 2 presents the characteristics of the DVT and pulmonary embolism (PE) in these 16 clinical cases. Among the 16 patients described with DVT, four also presented PE. Most patients had unilateral DVT (68,8%) and the proximal veins were more affected (75%). Drug thromboprophylaxis was performed in two patients: in case number 2, prophylactic enoxaparin was used; in case 6, acetylsalicylic acid (ASA) was used. In three patients, there were changes in the coagulation system, even without the use of medication thromboprophylaxis at the time of diagnosis of the thrombotic event.

Three patients presented risk factors commonly associated with thrombosis: case 2 was hospitalized for bacterial sepsis prior to the diagnosis of DVT; case 6 was a smoker and an injectable contraceptive user; and case 12 used cyclophosphamide to treat ENL.

Regarding the outcome of the cases, the majority (87.5%) did not present any complications. However, one patient remained with chronic lymphedema in one lower limb and another patient died whose cause was not identified.

Soon after the diagnosis of thrombosis, all were anticoagulated with enoxaparin and subsequently warfarin, except for the third clinical case described in this article for refusal of treatment.

**Figure 1:**
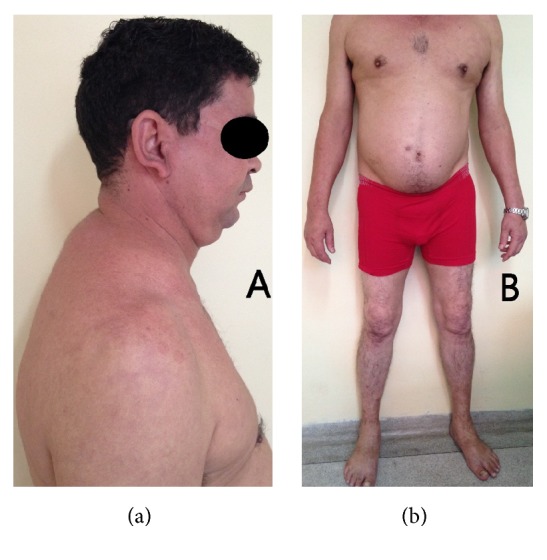
(a) Case 1: patient with Cushing's syndrome secondary to systemic corticosteroid; (b) Case 1: patient without edema after recovered DVT in right leg.

**Figure 2:**
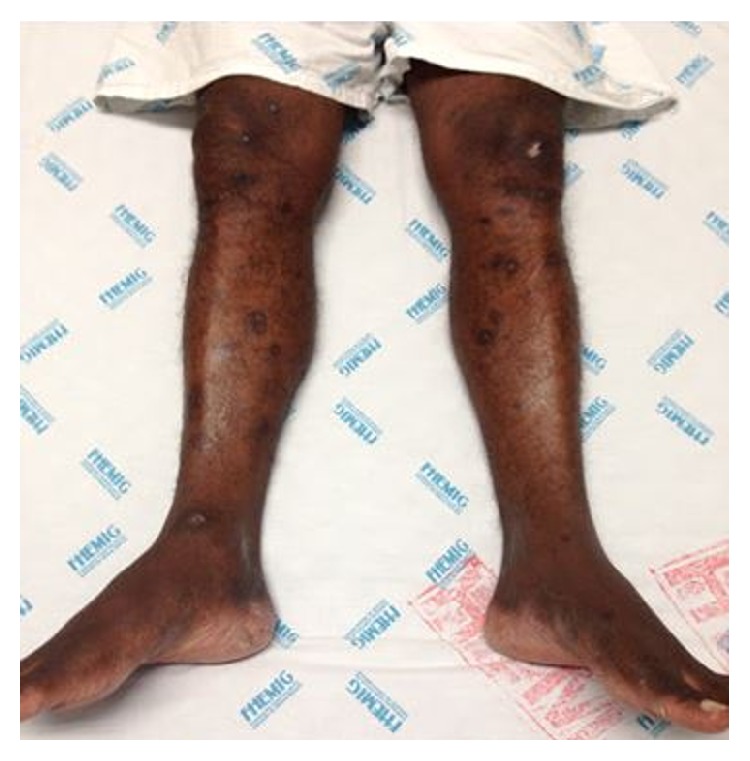
Case 2: patient without edema after recovered DVT in both legs.

**Figure 3:**
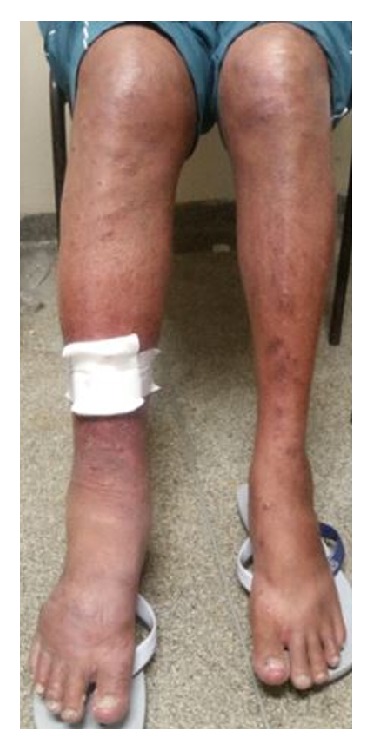
Case 4: patient with edema during acute DVT in right leg.

**Figure 4:**
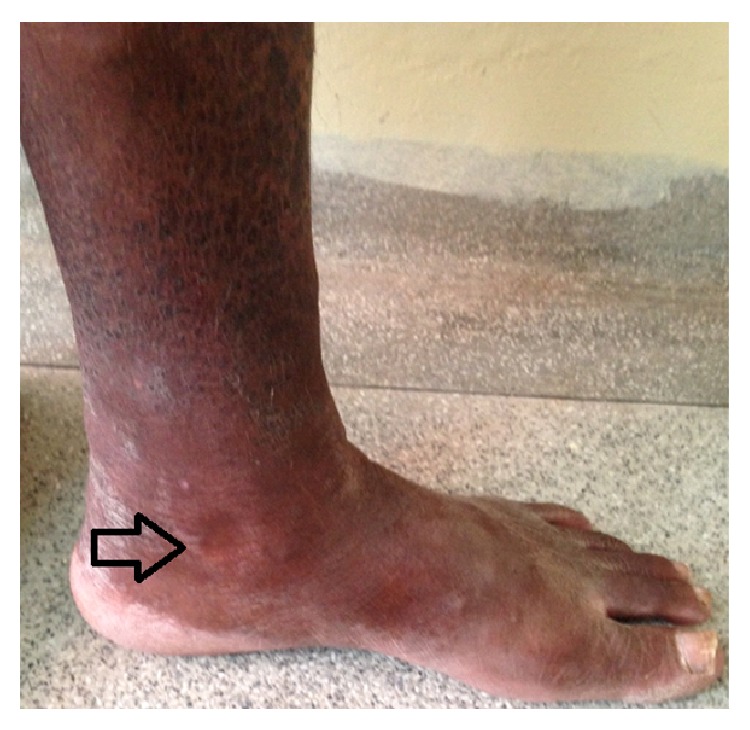
Case 5: patient with edema after recovered DVT in left leg.

**Figure 5:**
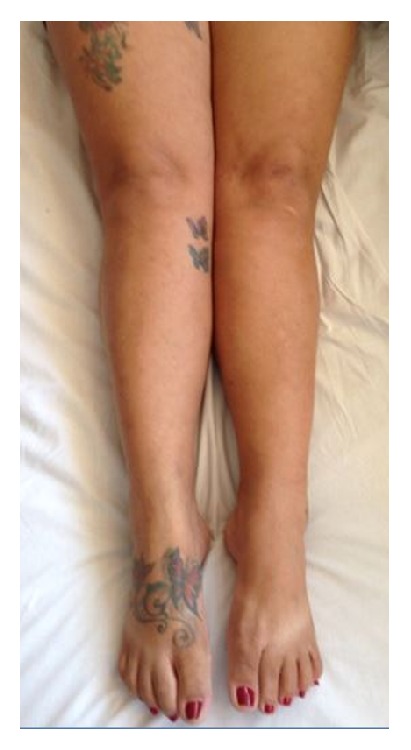
Case 6: patient without edema after recovered DVT in right leg.

There are five figures of the patients to illustrate the cases from HEM-FHEMIG.  Figure 1(a) is about case 1 with Cushing's syndrome secondary to systemic corticosteroid. Figures 1(b), 2, and 5 are about cases 1, 2, and 6 without edema of the legs after recovered DVT.  Figure 3 is about case 4 with edema during acute DVT in right leg.  Figure 4 is about case 5 with edema after recovered DVT in left leg.

Between 2014 and 2015, we followed 120 patients in the HEM-FHEMIG dermatology service using Thalidomide and oral corticosteroid for the treatment of ENL. In this period, we observed eight cases of DVT, which means an incidence of 6.66% of cases of thromboembolic events among those individuals.

## 5. Discussion

The drugs used in the treatment of ENL are thalidomide, oral corticosteroids, clofazimine, azathioprine, mycophenolate mofetila, methotrexate, and immunoglobulin G. Thalidomide is used in the treatment of ENL because of its anti-inflammatory property which is attributed to the inhibition of cytokines, TNF-alpha, endothelial growth factor, and fibroblast growth factor. The association of thalidomide with corticosteroid generates alterations in endothelial cell function, modifies the Th-1 cellular response, and increases the secretion of interferon-gamma and interleukin-2. The drug is also used in the treatment of multiple myeloma and renal carcinoma [8].

Thromboembolic events have been described in the association of thalidomide and corticosteroid in the treatment of multiple myeloma, but there are few reports when these drugs are used in the treatment of ENL. In addition, despite the existence of reports of prophylactic anticoagulation in individuals with multiple myeloma using the combination of thalidomide and corticosteroid, we did not find in the literature this prophylaxis for ENL [10].

Clinical suspicion of DVT should be confirmed by a complementary propaedeutic, in order to identify the involved vessel and the extent of thrombosis, since the risk of PE due to proximal DVT is greater, as well as the severity of chronic venous insufficiency [11, 16].

In studies with multiple myeloma patients, the incidence of DVT at the time of use of thalidomide alone is in the range of 2-3% and increases to 12-26% in the case of combination of thalidomide and dexamethasone without prophylactic anticoagulation, but the anticoagulation reduces the chances of DVT [17, 18].

In the results of this article, HEM-FHEMIG presented a 6.66% incidence of thrombosis in patients using Thalidomide for the treatment of ENL, which is more than twice as described in patients with multiple myeloma in use of thalidomide. We believe that this difference is due to the frequent association of corticosteroid and thalidomide in our patients.

In Brazil, the Ministry of Health recommends ASA 100mg/day as prophylaxis for DVT in case of combination of thalidomide and corticosteroid for the treatment of ENL [4, 19].

A study of adverse effects of thalidomide was performed in HEM-FHEMIG and DVT were recorded in the use of the association thalidomide and systemic corticosteroid in patients with ENL [20]. HEM-FHEMIG is a Reference Center of leprosy and is aware in order to ensure timely diagnosis, treatment and prevention of complications [21, 22].

The risk factors commonly associated with DVT are smoking, drug use, immobility, cancer, heart disease, inflammatory bowel disease, and previous history of thrombotic events [9].

The risk of DVT is related to genetic factors and is higher in blacks, intermediate in Caucasians, and lower in Asians. The incidence and prevalence of DVT are also strongly related to age, increasing almost 90-fold from 15 to 80 years of age. There is no definitive data on frequency between genders. Contraceptive use and HIV infection increase the incidence of DVT [23].

The increase in thromboembolic events in patients with ENL in treatment with thalidomide and corticosteroid is not related to genetic events of thrombophilia, but rather the association of the two medications [24].

## 6. Conclusion

The increased risk of thromboembolic events in ENL patients is not related to genetic events of thrombophilia and should be considered when prescribing the combination thalidomide and corticosteroids.

When this association is necessary, the use of ASA 100mg/day prophylaxis for DVT should be evaluated and the occurrence of DVT should be monitored to avoid complications.

## Figures and Tables

**Table 1:** Reports of ENL complicated by thrombosis during thalidomide/corticosteroids therapy.

N	COUNTRY	AGE (YEARS)	GENDER	TYPE OF LEPROSY	MDT	COMORBITIES	REFERENCE
1	BRAZIL	43	M	LL	No	No	CASE 1
2	BRAZIL	39	M	BL	Yes	Cutaneous leishmaniasis	CASE 2
3	BRAZIL	57	F	LL	MOC	No	CASE 3
4	BRAZIL	40	M	LL	No	HIV+	CASE 4
5	BRAZIL	54	M	LL	Yes	No	CASE 5
6	BRAZIL	34	F	LL	No	Diabetes, ex-alcoholic, asthma and mood disorder	CASE 6
7	BRAZIL	55	M	LL	No	No	CASE 7
8	BRAZIL	71	F	LL	No	HIV+ e diabetes	CASE 8
9	SPAIN	43	M	B	No	No	Petiti-Martin et al. 2013
10	ÍNDIA	35	M	LL	Yes	No	LLetricheLLLLel et al. 2008
11	USA	39	M	B	Yes	No	Fabi et al. 2009
12	ÍNDIA	37	F	MB	Yes	No	Sharma et al. 2004
13	PORTUGAL	33	M	B	Yes	HIV+	Medeiros et al. 2009
14	JAPAN	58	M	B	Yes	HL; HTN	Sayaka et al 201
15	BRAZIL	75	M	BL	No	No	Brito et al. 2010
16	SRI LANKA	60	M	LL	Yes	No	Ahamed et al 2011 9:2.

B, borderline leprosy; BL, borderline lepromatous leprosy; ENL, erythema nodosum leprosum; F, female; HL hyperlipidemia; HTN, hypertension; LL, lepromatous leprosy; M, male; MB, multibacillary; MDT, multi-drug therapy.

**Table 2:** Reports of DVT and PE.

N	DVT	PE	COAGULATION DISTURB	PHARMARCOLOGICAL PROPHLAXIS	RISK FACTOR TO DVT	OUTCOME AFTER DVT	REFERENCE
1	Unilateral proximal	No	No	No	No	Recovered	CASE 1
2	Bilateral proximal	No	No	Enoxaparina	Bacterial sepsis	Recovered	CASE 2
3	Bilateral proximal	No	No	No	No	Recovered	CASE 3
4	Unilateral proximal	No	No	No	No	Edema unilateral and death	CASE 4
5	Unilateral proximal	No	No	No	No	Edema unilateral	CASE 5
6	Unilateral distal	No	No	ASA	Smoking and medroxyprogesterone	Recovered	CASE 6
7	Unilateral distal	No	No	No	No	Recovered	CASE 7
8	Unilateral	No	No	No	No	Recovered	CASE 8
9	Unilateral proximal	No	Thrombophilia	No	No	Recovered	Petiti-Martin et al., 2013
10	Bilateral proximal	No	No	No	No	Recovered	LLetricheLLLLel et al., 2008
11	Unilateral proximal	Yes Bilateral	No	No	No	Recovered	Fabi et al., 2009
12	Unilateral proximal	No	No	No	Cyclophosphamide	Recovered	Sharma et al., 2004
13	Bilateral proximal	Yes	No	No	No	Recovered	Medeiros et al.,2009
14	Bilateral	Yes Bilateral	No	No	No	Recovered	Sayaka et al., 201
15	Unilateral proximal	No	Coagulogram disturb	No	No	Recovered	Brito et al., 2010
16	Unilateral proximal	Yes Bilateral	Thrombocytopenia 90.000	No	No	Recovered	Ahamed et al., 2011 9:2.

DVT, deep vein thrombosis; PE, pulmonary embolism; ASA, acetyl salicylic acid.
